# Targeting the Barriers Driving Immune Exclusion

**DOI:** 10.3390/ph19071012

**Published:** 2026-06-30

**Authors:** Sofia Alvarez-Lorenzo, Inés Velázquez-Quesada, Marco Antonio Velasco-Velázquez

**Affiliations:** 1 School of Medicine, Universidad Nacional Autónoma de México, Mexico City 04510, Mexico; sofi.alvarez1707@gmail.com; 2 Bioengineering Department, Temple University, Philadelphia, PA 19122, USA; ines.velazquez.quesada@temple.edu

**Keywords:** immune exclusion, immune infiltration, tumor microenvironment, cancer immunotherapy, extracellular matrix

## Abstract

Immune exclusion refers to the phenomenon in which immune cells are restricted to the peritumoral stroma. This phenomenon arises from complex interactions within the tumor microenvironment (TME) that limit immune cell infiltration. Consequently, immune exclusion represents a major barrier to effective antitumor immunity and a key obstacle to the success of immunotherapy. The principal components of the TME that orchestrate immune exclusion are (i) the tumor vasculature, (ii) the extracellular matrix (ECM), and (iii) stromal cells and their chemokine-mediated signaling. Understanding immune exclusion is critical for designing therapies that enhance the efficacy of immunotherapy and improve clinical outcomes. This review synthesizes current knowledge of the molecular and cellular mechanisms underlying immune exclusion and discusses emerging therapeutic strategies aimed at overcoming this phenomenon.

## 1. Introduction

Immunotherapy has improved the clinical outcome of cancer patients by restoring the antitumor immune response in multiple types of cancer, including melanoma, breast cancer, lung cancer, gastric cancer, colorectal cancer, and Hodgkin lymphoma, among others [1]. The two principal immunotherapeutic approaches to treat cancer are (i) immune checkpoint blockade (ICB), which inhibits the suppression of T cell activation by targeting checkpoints such as CTLA-4 or PD-1/PD-L1; and (ii) adoptive cell therapy (ACT), by using ex vivo engineered T cells engineered with a chimeric antigen receptor (CAR) that enables them to identify specific targets through an antibody-derived binding domain [2,3]. As of January of 2026, there are nine FDA-approved ICB drugs and seven approved ACT drugs [4], meaning that immunotherapy has now become a cornerstone of cancer therapy. However, immunotherapy elicits a highly variable efficacy among cancer patients. Response rates range from 10 to 60% in solid tumors treated with ICB drugs and 54 to 77% in hematological malignancies receiving ACT [5,6,7,8,9].

Even when there is room for improvement in toxicity and costs, the main obstacle for wide use of immunotherapy is its efficacy [10]. Thus, immunotherapy is not the first-line treatment for most cancer patients. The identification of the mechanisms hindering clinical efficacy may lead to the development of new adjuvant therapies that improve the efficacy of immunotherapy. Those combined therapies could generate complete and durable responses, or even achieve curative outcomes, as has been reported for immunotherapy in hematologic tumors and melanoma [10].

A key element influencing immunotherapy response is the tumor microenvironment (TME), which can function as a barrier for immune effector cells. Solid tumors can be classified into three main phenotypes, based on their degree of immune infiltration: (i) immune-inflamed, (ii) immune-desert, and (iii) immune-excluded [2,11]. Immune-inflamed tumors, also known as “hot” tumors, have a high infiltration of tumor-infiltrating lymphocytes (TILs), including CD4^+^ and CD8^+^ T cells, myeloid, and monocytic cells [2,11]. Those tumors show a proinflammatory milieu and immunogenicity driven by genomic instability and frequently display PD-L1 expression [2,11]. It has been hypothesized that the presence of these immune components is a sign of a previous immune response, displaying tumors that generally respond well to immunotherapy [2,11]. Solid tumors with a high tumor mutational burden show a good response to ICB and have a better prognosis [2]. Immune-desert or “cold” tumors have either an absence or very low number of TILs; they have immunosuppressive response, with limited inflammatory cues, poor antigen presentation and low expression of immune checkpoints. Those characteristics render immune-desert tumors unresponsive to immunotherapy [2]. Immune-excluded tumors are characterized by abundant leukocytes within the surrounding stroma that are unable to penetrate the tumor parenchyma. In such a subset of tumors, the TME is different than in other tumor phenotypes, shaped by particular chemokine profiles, vascular barriers, and stroma components [11]. For example, the stroma can “encapsulate” the tumor, and it can even penetrate it, creating the impression that immune cells are present within the tumor, even though they have not truly accessed the tumor tissue [11]. The immune-excluded phenotype is not associated with a particular type of cancer, as it is present in multiple cancer types, such as pancreatic cancer, colorectal cancer, breast cancer, ovarian cancer or lung cancer, and they can account for 19–75% of the tumors in patients [2] (Table 1). Identification and analysis of immune-excluded tumors can be achieved by multiple methods, each one with specific advantages/disadvantages and clinical usefulness (Table 2).

Patients with immune-excluded tumors do not respond to immunotherapy, have a worse prognosis, and are more likely to experience recurrence compared to both inflamed and even immune-desert tumors [17,18,19]. For example, treatment with ICB agents may trigger T cell activation and proliferation, but in immune-excluded tumors they are unable to infiltrate and cannot kill cancer cells [2,11]. Accordingly, T cell infiltration has been pointed out as the critical bottleneck for the efficacy of immunotherapies [2,11]. Therefore, elucidating the molecular and cellular mechanisms underlying immune exclusion is essential for the development of new strategies aiming to allow the infiltration of immune cells.

Herein, we explore the composition of the TME in immune-excluded tumors, focusing on the key elements that constitute the barriers that prevent infiltration. We also highlight emerging therapeutic targets and novel therapeutic strategies designed to reprogram the immune-excluded milieu, aimed at converting immune-excluded tumors into inflamed phenotypes for improved response to immunotherapy.

**Table 2:** Methodological approaches for the identification and characterization of immune-excluded tumors.

Method	Goal	Advantages	Disadvantages	Applicability
Immunohistochemistry (IHC) [20]	Analysis of differential immune cell distribution across tumor regions	Compatible with biopsy or surgical specimens; cost-effective and widely adopted	Non-standardized immune-exclusion criteria; limited multiplex marker analysis	Clinical routine
Multiplex immunohistochemistry (mIHC) or immunofluorescence (mIF) [21]	Spatial and molecular profiling of immune cells	Compatible with clinical specimens	Needs automated platforms, antibody validation, and specialized analysis software	Translational/limited clinical use
Spatial transcriptomics [22]	Spatial and molecular profiling of immune cells	Multiplex transcript detection while preserving spatial context	High cost and analytical complexity	Translational and preclinical research
RNA sequencing (RNA-seq) [23]	Detection of transcriptional programs associated with immune exclusion	Transcriptomic profiling and pathway activation	Loss of spatial information	Translational/limited clinical use
Single-cell RNA sequencing (scRNA-seq) [20]	Identification and quantification of cellular states and transcriptional signatures	Resolution of cell-specific gene expression and heterogeneity	High cost, analytical complexity, and loss of spatial context	Translational and preclinical research
Machine learning analysis of whole-slide images [24]	Analysis of immune cell spatial organization and tissue architecture	Quantitative, reproducible and fast spatial immune profiling	Requires extensive benchmarking	Translational/limited clinical use

## 2. Immune Exclusion Employs Physical and Chemical Barriers

A common characteristic of clinical-relevant tumors is that they have gone through immunoediting, meaning that the cancer cell clones are shaped to survive the host immune response. However, the mechanisms and the extent of immune evasion are distinct. Immune-excluded tumors establish barriers that block the infiltration of immune cells. Those barriers, which cooperate with other mechanisms for immune evasion, include (i) dysfunctional tumor vasculature that provides structural impediment for immune cell infiltration, (ii) extracellular matrix (ECM) with alterations in its composition and biomechanical properties, and (iii) the presence of subsets of stromal cells—such as cancer-associated fibroblasts (CAFs)—that produce immunosuppressive mediators. Altogether, these elements impede cytotoxic T cell infiltration, driving poor clinical outcomes. The disruption of those barriers could restore immune infiltration and enhance the efficacy of existing immunotherapies. Thus, the different components of the barriers driving immune exclusion are therapeutic targets that may open new avenues for combinatorial treatment strategies.

### 2.1. Tumor Vasculature

The formation of new vasculature is a tightly regulated process governed by a balance in stimulating and inhibiting factors [17]. During tumor progression, this balance is disrupted by the overexpression and release of growth factors and cytokines. The newly formed tumor blood vessels are abnormal, disorganized, and functionally impaired [17,18]. Two distinct mechanisms contribute to tumor vascularization: angiogenesis and vasculogenesis [17,18]. Angiogenesis refers to the formation of new blood vessels from existing vasculature and is regulated by a network of signaling molecules, including proangiogenic (i.e., vascular endothelial growth factors (VEGFs), fibroblast growth factors (FGFs), and platelet-derived growth factor-B (PDGF-B)) and antiangiogenic factors (i.e., endostatin and angiostatin) [17]. Vasculogenesis involves the de novo formation of blood vessels through the recruitment of endothelial progenitor cells and pericyte precursors, which are attracted to the tumor by growth factors and interleukins [18]. The dysfunctional vasculature found in tumors represents a structural impediment for immune cell infiltration and is therefore associated with T cell exclusion (reviewed by [25,26,27]). Two main mechanisms have been described: endothelial anergy and alterations in the endothelial basement membrane (E-BM).

First, soluble factors released during angiogenesis and vasculogenesis actively alter the response to inflammatory cytokines and, consequently, the expression of adhesion molecules on the tumor endothelium cells [19,28,29]. The disruption of the interaction between endothelial and immune cells, defined as endothelial anergy, impairs immune cell recruitment [29]. Well-documented examples are the downregulation of E-selectin, P-selectin, and the integrin ligands VCAM1, ICAM1, and MAdCAM-1 on endothelial cells [30,31,32,33]. Endothelial growth factors (VEGFs and FGFs) are responsible for the reduction in the expression of the listed adhesion molecules, as they inhibit the transcriptional upregulation generated by tumor necrosis factor-α (TNF-α) and IL-1β [34,35]. The low expression of adhesion molecules suppresses the interaction of immune cells, such as T cells, with the vessel wall; consequently, the cells cannot migrate across the endothelial barrier, preventing their entry into the tumor parenchyma [19,28]. Endothelial anergy has been reported in an array of different tumor types, such as hepatocellular carcinoma [30], melanoma [32] and others [31,36].

In renal cell carcinoma (RCC) patients, treatment with the antiangiogenic drugs Sunitinib or Bevacizumab reverses tumor endothelial anergy, leading to a more inflammatory tumor microenvironment. The effect is marked by increased ICAM-1 levels and increased infiltration of major leukocyte populations (T cells, macrophages, and dendritic cells) [37]. In vitro analyses confirmed that the antiangiogenic drugs restored normal ICAM-1 expression on angiogenic endothelial cells, and various tyrosine kinase inhibitors promoted transendothelial migration of non-adherent and monocytic leukocytes [37]. In agreement, combining FGFR blockade with immune checkpoint therapy improves antitumor efficacy. Treatment with an anti-VEGF antibody (B20) enhances the infiltration and distribution of EGFRvIII-targeted CAR-T cells within the glioblastoma tumor microenvironment, delaying tumor growth and improving survival in glioblastoma-bearing mice compared to CAR-T therapy alone [38].

A recently described mechanism by which tumor vasculature can disrupt T cell infiltration is the alteration in the E-BM composition. Superoxide Dismutase 3 (SOD3) is an extracellular enzyme that participates in the redox homeostasis of the ECM and stimulates lymphocyte transmigration. In endothelial cells, SOD3 expression and activity induce transcriptomic changes in genes target of the HIF-2α [39,40] and canonical and non-canonical NF-κB pathways [40]. Consequently, SOD3 controls T cell extravasation through activating the transcription of VE-cadherin [39] and the E-BM component laminin-α4 [40]. High SOD3 levels are associated with human colon cancer infiltration by CD8^+^ T cells [40].

In pancreatic and lung cancer, SOD3 expression is reduced [31,32,41], suggesting that SOD3 activity is unfavorable for tumor progression. The reduced expression of SOD3 in lung and thymoma cancer models leads to decreased laminin-α4 levels in the E-BM, rendering its composition less permissive to T cell migration [39]. Whether SOD3 is playing the same role in immune exclusion across different cancer types and what additional mechanisms cooperate to such effect remain to be determined.

### 2.2. Extracellular Matrix (ECM)

The ECM is a highly organized and dynamic structure that provides tissue integrity and regulates biomechanical and biochemical signaling in normal tissues. The ECM can be separated into two main elements: (i) the interstitial matrix, which envelops the cells and is made up of fibrillar structures (mainly type I and III collagens), and non-fibrillar components (non-fibrillar collagens, glycoproteins, and proteoglycans) [42,43]; and (ii) the basement membrane, which is made up of type IV collagen, laminin, and perlecan, and lies between parenchyma and connective tissue [42]. In tumor tissue, the ECM is generated by multiple cell types, including fibroblasts, endothelial cells, macrophages, and cancer cells. The ECM serves as a key component of the TME and undergoes significant changes in its composition and structure that promote cancer progression and metastasis [44,45]. For example, breast cancer cells escape from dormancy at the metastatic site by inducing a switch in the expression of ECM components, which is elicited by subpopulations of cancer cells, proinflammatory stimuli, or the bidirectional communication between cancer cells and resident macrophages [46,47].

The biomechanical properties of the ECM are highly complex and play a critical role in regulating biological processes and cell behavior, including proliferation, migration, and immune exclusion. Alterations in biomechanical properties of tumoral ECM have been reported, such as stiffness, and fiber orientation and alignment [48,49]. ECM stiffness is the ability of resisting deformation in response to an applied force and depends primarily on the organization and density of collagen fibers [50]. A stiffer ECM typically contains a higher concentration of crosslinked collagen and reduced pore size, which can limit cell migration. In contrast, a less stiff ECM has a lower density of crosslinked collagen fibers and larger pores, allowing for increased cellular movement [50]. The degree of stiffness depends also on enzyme activity. For example, lysyl oxidases (LOX), lysyl hydroxylases, and transglutaminases increase ECM stiffness by promoting protein crosslinking, while matrix metalloproteinases (MMPs) regulate both ECM synthesis and degradation [51].

LOX enzymes are amine oxidases dependent on copper expressed in a variety of cell types. They are responsible for the initiation of the crosslinking of collagens and elastin, having a very important role in the ECM, in homeostasis, and in diseases such as cirrhosis, atherosclerosis, and cancer [52]. In colon cancer, LOXL2 promotes ECM remodeling and alignment, creating a dense physical barrier that restricts CD8^+^ T cell infiltration into tumor nests and contributes to immune exclusion [53]. Inhibition of LOXL2 enhances T cell infiltration and improves response to immunotherapy. Mechanistically, GPR4 regulates LOXL2 through the JAK2/STAT3 pathway and increases collagen I deposition via TGF-*β* signaling, further reinforcing the collagen-mediated barrier within the tumor microenvironment. The listed findings were validated in colon cancer cell lines, in vivo models and cancer patient samples [54].

LOXL1 is also overexpressed in colorectal cancer and is associated with poor tumor differentiation and worse prognosis. It promotes tumor progression by enhancing proliferation, migration, invasion, and driving epithelial–mesenchymal transition (EMT), while also contributing to an immunosuppressive tumor phenotype. High LOXL1 expression correlates with reduced CD8^+^ T cell infiltration and poorer responses to immunotherapy, findings that were consistently validated across multiple CRC cohorts, cell lines, and clinical specimens [53]. In agreement, LOX family members promote glioma progression by fostering immune suppression, which is associated with increased infiltration of macrophages (particularly M2-like), eosinophils, neutrophils, Th2 cells, and dendritic cells, alongside reduced CD8^+^ T cell presence. Silencing LOX family members (LOX and LOXL1–4) impairs glioma cell proliferation, induces apoptosis, and reshapes immune cell behavior by reducing M2 macrophage polarization and enhancing CD8^+^ T cell activity [55]. Silencing LOX family genes significantly decreases CD8^+^ T cell apoptosis, increases IFN-γ and TNF-α production, and improves cytotoxic function in vitro. In a xenograft model, LOX suppression enhances antitumor immunity, elevating IFN-γ and TNF-α levels in tumor tissues and reducing tumor growth [55]. Overall, LOX family members promote glioma progression by promoting immune evasion, while their inhibition restores antitumor immune responses through reduced M2 polarization and enhanced CD8^+^ T cell activity [55]. This evidence indicates that LOX enzymes can be targeted in glioma for partial restoration of immune responses.

Mechanosensors can also participate in ECM remodeling. Piezo1 is a mechanosensitive ion channel that regulates changes in ECM stiffness. In cancer cells, Piezo1 activation reinforces mechanosignaling, creating a self-sustaining cycle that supports tumor progression [56]. Modulation of Piezo1 signaling, particularly in combination with matrix normalization, can enhance the infiltration and antitumor efficacy of the immune response [57]. Thus, targeting Piezo1 may offer a promising therapeutic strategy, which has been reviewed elsewhere [56,58,59].

As increased density in the ECM fibers restricts the movement of T cells, they accumulate in the stroma [44,45]. For example, a high density of collagen is related to poor prognosis in tumors [42], highlighting its participation in cancer biology. The expression of collagen I in breast cancer and that of collagen III in colorectal cancer have been closely linked to tumor progression [60,61]. Beyond collagen abundance, its architecture plays a critical role in cancer progression. Cancer cells can induce changes in collagen deposition that may result in dense, crosslinked and disorganized fibers that do not allow cell immune penetration. For example, the binding of discoidin domain receptor 1 (DDR1) to collagen activates multiple signaling pathways [62] that cooperate to induce an ECM remodeling, consisting of the alignment of collagen fibers, which blocks immune cell infiltration in breast cancer mice models (E0771, M-Wnt and AT-3 cells) [63]. In TNBC human samples, DDR1 correlates negatively with TIL levels [63]. Furthermore, the KO of *Ddr1* increases the infiltration of T cells into the tumor and decreases tumor growth in mice models [63]. Sun et al. showed that the extracellular domain (ECD) of DDR1 is responsible for immune exclusion, and ECD-neutralizing antibodies disturb the alignment of collagen fibers, inhibit tumor growth, and increase immune cell infiltration [63]. Disorganized collagen structure has also been reported in vestibular schwannoma, where the heterogeneous collagen deposition acts as a physical barrier to immune cell infiltration and alters the biomechanical properties of the TME [64].

Heparan sulfate proteoglycans (HSPGs) are essential constituents of the ECM. They contribute to both tumor progression and tumor exclusion through modulation of chemokine and growth factor signaling, as well as interference with immune cell infiltration by swelling the physical density of the ECM [43]. However, the mechanism by which heparan sulfate proteoglycans exactly influence cell infiltration remains unclear. The degradation of heparan sulfate proteoglycans by heparanase (HPSE) improves tumor infiltration and antitumor activity of CAR-redirected T lymphocytes in breast cancer models [65], supporting the role of heparan sulfate proteoglycans in immune exclusion.

HPSE is expressed in an array of immune cells such as lymphocytes, NK cells, DCs, macrophages, and neutrophils, among others [66]. In innate immune cells such as NK cells, HPSE activity is essential for effective invasion into tumor tissue through the ECM, thereby facilitating immune surveillance [66]. However, in murine pancreatic cancer models, tumor cell overexpression of HPSE increased macrophage infiltration and tumor volume compared to tumors with normal HPSE expression [67]. Notably, these macrophages exhibited polarization toward an M2 phenotype, highlighting the context-dependent and potentially controversial role of HPSE in tumor immunity [67]. Together, these findings emphasize the importance of understanding the mechanisms by which HSPGs and their degradation by HPSE regulate immune cell function within the tumor microenvironment, as it may increase T cell trafficking while promoting an immunosuppressive myeloid microenvironment.

Hyaluronan (HA) is a major glycosaminoglycan of the ECM. HA regulates an array of processes like cell growth, adhesion, migration, and it can also regulate immune responses through the interaction with CD44, TLR4, and proteoglycans in a molecular-weight-dependent manner [68,69]. Hyaluronidase enzymes such as HYAL1-3 and PH20 convert high-molecular-weight (HMW)-HA to low-molecular-weight (LMW)-HA. HA is overexpressed in solid tumors, and its abundance is related to worse prognosis [70]. Pharmacological depletion of HA by PEGylated human hyaluronidase (PEGPH20) treatment reduces tumor growth and remodels the stroma in colon cancer models, with reduction in Treg and myeloid cells, and increased infiltration of CD8^+^ T cells within the tumor [71]. scRNA-seq of those tumors showed that HA degradation induces strong stroma remodeling driven by multidirectional communication between immune and stromal cells, providing relevant information about the molecular mechanism by which HA induces cell exclusion [71].

In a pancreatic cancer murine model, HA accumulation restricted cell numbers of CD4^+^, CD8^+^ and NK cells within the tumor [72]. In analysis from samples from pancreatic cancer patients, stromal HA expression was associated with low immune score and response, as well as poor survival [70]. In agreement, HA-positive mesenchymal colorectal cancer (mCRC) patients have a worse prognosis than those with low levels of HA [71]. In colon tumors rich in HA, HA degradation induced by a fusion protein (TAVO423) containing hyaluronidase and a bispecific antibody targeting CAF remodels the TME facilitating TILs infiltration within the tumor and enhances tumor growth inhibition [73]. The combination of TAVO423 with anti-PD-1, T cell engagers or ADCs increases T cell infiltration and improves response to immunotherapy in colon and pancreatic tumors [73].

### 2.3. Stromal Cells and Chemokine Secretion

Cells in the stroma secrete factors that support tumor progression or generate an immunosuppressive environment. Increased numbers of CAFs and myeloid-derived suppressor cells (MDCS) on the TME have been associated with poor prognosis and unresponsiveness to immunotherapy [74,75,76,77,78]. For a review covering the relevance of these cells in immunosuppression, see [79].

CAFs participate in the production of the ECM components as well as in the regulation of its stiffness [80]. For example, TGF-*β* can stimulate the production of collagen by CAFs and increase collagen crosslinking by increasing the expression of the enzymes involved in this process, such as the LOX family [80]. In metastatic urothelial cancer, patients with active TGF-*β* signaling show tumor-excluded phenotype, because of a stiff environment rich in fibroblasts and collagen that encapsulates the tumor. Furthermore, TGF-*β* signaling correlates with a lack of response to ICB [74,75,81,82], indicating clinical relevance in therapeutic responses.

CAFs are also responsible for producing the ECM protein periostin (POSTN), which mediates cellular adhesion and tissue repair by interacting with α_v_-containing integrins. POSTN is upregulated in tumor tissue [15,81,83,84]. In samples from TNBC patients, periostin is overexpressed in immune-excluded and stroma-rich tumors, where it is associated with a worse recurrence-free survival and a reduction in TILs [15]. In gastric cancer, POSTN expression is associated with ICB resistance [83]. In hepatocellular carcinoma, POSTN^+^ CAFs act as barriers to immune response, decreasing T cell infiltration and immunotherapy efficacy. Patients with higher levels of POSTN^+^ CAFs had worse responses to ICB [84]. Even when this evidence suggests that periostin cooperates in immune exclusion, additional research is needed to clarify its role. At present, no therapies targeting POSTN or POSTN^+^ CAFs have been developed.

Beyond altering the biophysical properties of ECM, stromal cells cooperate with tumor cells in secreting chemical signals that promote infiltration disruption. Chemokines and their receptors are crucial for T cell infiltration. For example, CXCL9 and CXCL10 participate in T cell tumor recruitment, as tumors with high expression exhibit greater CD8^+^ T cell numbers and a better response to immunotherapy, in contrast to those tumors with low levels of those chemokines [25]. These two chemokines (alongside CCL4 and CCL5) are included in a conserved 4-chemokine signature mark proposed by Romero and colleagues for resectable and metastatic pancreatic adenocarcinoma tumors with an active antitumor phenotype [85]. The expression of such a signature positively correlates with transcriptional metrics of T cell activation, increased T cell activation scores, and active signaling of T cell priming, consistent with an inflamed phenotype [85]. Accordingly, dendritic cells engineered to express CXCL9 and CXCL10 (CXCL9/10-DC) inhibit tumor growth while promoting robust T cell infiltration and activation within the tumor microenvironment in murine NSCLC models [86,87]. The antitumor effect of CXCL9/10-DC relies on both CD4^+^ and CD8^+^ T cells, as well as CXCR3-mediated trafficking of T cells from lymph nodes [86]. Furthermore, the combination of CXCL9/10-DC with anti-PD-1 therapy overcomes ICB resistance and generates durable tumor-specific immune memory [86].

On the contrary, the CXCL12/CXCR4 axis attracts T cells to regions rich in CXCL12, which is also surrounded by CAFs and ECM, confining T cells and disrupting their infiltration to the tumor [85,88]. In pancreatic, colorectal, and breast cancer, tumor cells are coated with CXCL12–keratin-19 (KRT19) heterodimers organized into filamentous networks generated by the enzyme transglutaminase 2. This CXCL12–KRT19 coating stimulates CXCR4 but reduces T cell motility as dimerized CXCL12 suppresses migration [89]. Thus, tumors coated with CXCL12–KRT19 are resistant to anti-PD-1 therapy. On the other hand, tumors lacking KRT19 or the CXCL12–KRT19 coating display increased infiltration of activated CD8^+^ T cells and enhanced sensitivity to immunotherapy [89].

These findings indicate that chemokines modulate T cell infiltration and antitumor T cell responses. Notably, aberrant chemokine distribution within the ECM may prevent effective T cell–tumor cell interactions, even after T cell infiltration following ECM disruption.

## 3. Overcoming Tumor Immune Exclusion: Therapeutic Strategies

### 3.1. Targeting the Tumor Vasculature

Therapies targeting the tumor vasculature aim to modulate key signaling pathways within the TME. For example, the delivery of low doses of TNF-α via a vascular-targeted fusion protein (NGF-TNF) activated endothelial cells and increased the number of TILs [19]. Thus, combining NGF-TNF with ICB or ACT enhanced T cell extravasation and effector functions, ultimately improving survival in murine tumor models [90,91].

Tumor endothelium is normally “anergic” due to high levels of VEGF. Thus, strategies that target VEGF have been combined with engineered T cells, which require tumor infiltration for their therapeutic effect. Supper et al. developed CAR-T cells engineered to secrete a VEGF-targeting single-chain variable fragment. In metastatic murine models of ovarian and lung cancer, those cells simultaneously inhibited angiogenesis and improved CAR-T cell migration into the tumor. This dual approach significantly improved tumor control and outperformed treatment with standard anti-VEGF plus CAR-T [92]. In a different strategy, combined administration of ex vivo engineered T cells armed with tumor-targeting antibodies and anti-VEGF/VEGFR2 agents efficiently reduces tumor volume and induces T cell infiltration in murine models of neuroblastoma and osteosarcoma [93]. These findings show that targeted modulation of VEGF signaling enhances T cell functionality by facilitating their infiltration and intratumoral distribution.

Given its role in controlling the phenotype of tumor endothelial cells and E-BM, SOD3 may become a therapeutic target. Some indirect evidence suggests that the modulation of SOD3 may impact the vasculature’s function in tumors. SOD3 upregulation in the TME, induced either by genetic forced re-expression or by the antihypercholesterolemic drug lovastatin, increases chemotherapy delivery and effectiveness on Lewis lung adenocarcinoma (LLC) tumors [94]. SOD3 expression can also be induced by Farnesoid X receptor (FXR) activation, as the SOD3 gene promoter has an FXR-responsive element [95]. Thus, the combination of the FXR agonists GW4064 with an anti-PD-L1 antibody in a murine model of colorectal cancer improved the response by multiple mechanisms, including enhanced recruitment of CD4^+^ and CD8^+^ T cells into tumor sites [96]. Those results indicate that increased permeability in the tumor vasculature can be achieved and suggest that SOD3 re-expression may impact immune cell infiltration. However, lovastatin and FXR agonists have pleiotropic effects; thus, specific activators of SOD3 transcription or enzymatic activity are required to corroborate that pharmacological targeting of SOD3 can improve the response to immunotherapeutics.

The above examples validate the tumor vasculature as a promising target to overcome immune exclusion in solid tumors, which can, in turn, help overcome resistance to immunotherapy.

### 3.2. Targeting the ECM

Given the key role of collagen in controlling the stiffness of the ECM, the treatment with collagenase enhances the capacity of T cells to migrate into the tumor. For example, in an ex vivo model of human lung cancer, tumor slices from patients were locally treated with collagenase, which partially degraded the dense collagen network surrounding tumor islets and significantly increased the number of preactivated T cells able to contact tumor cells at the tumor–stroma interface [45]. Furthermore, the study showed that T cell trajectories are guided by aligned fibers around blood vessels and tumor regions, corroborating that the stromal ECM shapes antitumor immunity by controlling T cell positioning and migration [45]. To target collagen organization in TME, Liu et al. [97]. developed PRTH-101, a monoclonal antibody against DDR1 (see Section 2.2 above). Collagen binding to DDR1 triggers downstream signaling and promotes receptor shedding, a process linked to immune exclusion and tumor progression. By blocking DDR1, its shedding is inhibited, disrupting the DDR1/collagen axis and therefore promoting immune cell infiltration by reorganizing collagen fibers to create gaps in the tumor barrier [97]. Currently, the clinical potential of PRTH-101 is being evaluated in a Phase 1 trial (NCT05753722), testing it as monotherapy and as combination therapy with pembrolizumab in patients with metastatic solid tumors [98].

A different strategy with therapeutic potential is LOX inhibition. In mice, LOX inhibition through beta-aminopropionitrile reduced ECM stiffness, as measured by shear wave elastography and microscopy [99]. As a result, T cell migration was enhanced, improving the efficacy of anti-PD-1 therapy in pancreatic adenocarcinoma models [99]. However, in breast cancer models this effect was less important due to differences in the TME and ECM composition, with lower and dispersed stromal content. Furthermore, collagen fiber curvature (a property associated with a relaxed state of collagen fibers) was altered in all models, but significant changes in orientation were only observed in pancreatic models [99]. Importantly, authors inhibited LOX in early tumor stages, simulating a preventive setting, as their hypothesis was that LOX activity is higher at the initial stages of the tumor, when the fibrous stroma is in construction [99].

Similarly to what has been found for collagenases, other ECM-hydrolyzing enzymes successfully remodel the TME in murine models and have reached clinical evaluations. The most prominent example is the induction of HA degradation by hyaluronidase. In a breast cancer model, Farrera-Sal et al. combined an adenovirus expressing a T cell engager with a soluble version of human hyaluronidase (PH20) [100]. The treatment increases antitumor efficacy with more infiltration and higher T cell numbers, showing that the degradation of HA leads to increased T cell infiltration [100]. Furthermore, PH20 potentiates the antitumor activity of CAR-T cells by enhancing their transmigration and infiltration into tumors, leading to lower tumor burden in gastric cancer models [101]. A similar effect was observed in a pancreatic tumor model, where treatment with recombinant human hyaluronidase restored antitumor activity by the increase in TILs and NK cells numbers, inhibiting tumor growth [102]. In lymphoma and colon cancer mice models, the administration of CAR-T cells modified to express hyaluronidase and an anti-PD-L1 on their surface, improves cell biodistribution, enhances tumor penetration by degradation of the ECM, and displays strong antitumor effect [101]. Currently, multiple clinical trials evaluate the effect of hyaluronidase on cancer patients (Table 3). Noteworthy, PH20 has been well tolerated in advanced solid tumors, where it induced increased tumor perfusion and reduced tumor metabolic activity, indicating antitumor effect and supporting further evaluation [101,103].

The enzyme HPSE degrades syndecan-1, facilitating immune cell migration and enhancing antitumor activity [43]. CAR-T cells engineered to express HPSE had a higher capacity to infiltrate the tumor by degrading the ECM, having a higher antitumor activity [65]. As another example, NK cells expressing HPSE have demonstrated the ability to infiltrate tumors and eliminate cancer cells in both three-dimensional cultures and murine models [105]. These findings highlight a potential novel therapeutic strategy based on the use of engineered cells engineered to express HPSE.

The use of experimental therapies targeting the ECM has been further developed by engineering bacteria to be used as vectors. For instance, Yao et al. [108] developed a probiotic-nanosystem by conjugating *Clostridium butyricum* with Vactosertib-loaded liposomes via an MMP2-responsive peptide. Vactosertib is delivered by this nanosystem and inhibits the development of ECM, which results in a loosened ECM that allows deeper penetration of drugs and increases the tumor infiltration of effector immune cells. This approach reduced fibrinogen and collagen-I levels, decreasing ECM stiffness and enhancing immune cell infiltration [108]. Similarly, Li and Ye engineered an *E. coli* strain to secrete collagenase, which degraded tumor-associated collagen, suppressed tumor growth, and improved survival in murine models [104]. The use of this enzyme-delivery strategy that facilitates immune cell infiltration and exhibits synergistic effects with chemotherapy has proved their therapeutic potential [104,108,109,110].

Collectively, these findings demonstrate that the ECM can be targeted to modulate immune exclusion and antitumor immunity. Therapeutic strategies aimed at remodeling the ECM—including collagen degradation, inhibition of collagen organization and crosslinking, degradation of HA, enzymatic targeting of proteoglycans, and the use of engineered immune cells or bacteria to locally deliver ECM-modifying agents—consistently enhance immune cell infiltration and improve antitumor responses in preclinical models. Importantly, several of these approaches have progressed to clinical evaluation (Table 3), highlighting their translational potential. Nevertheless, the heterogeneous responses observed across tumor types indicate that ECM composition, architecture, and biomechanical properties critically influence therapeutic efficacy. Therefore, the identification of predictive biomarkers and the optimization of treatment timing will likely be essential for the successful implementation of ECM-targeting therapies. Moreover, while ECM-targeting approaches may enhance immune cell infiltration and improve the response to therapy, ECM degradation and remodeling are known contributors to metastatic dissemination and may even promote immunosuppressive niches, as reviewed elsewhere [111,112,113]. Thus, therapy-induced ECM alterations can sometimes promote tumor progression mechanisms. Therefore, strategies aimed at overcoming immune exclusion should ideally promote ECM normalization rather than indiscriminate matrix degradation, thereby improving immune access while preserving tissue integrity and limiting protumoral effects.

### 3.3. Targeting Stromal Cells and Chemokine Signaling

Targeting CAFs has proven highly effective in impeding tumor progression. This can be achieved by inhibiting CAFs receptors or targeting surface proteins. For instance, in an orthotopic breast tumor mice model, blockage of Endo180, a fibroblast receptor that binds collagen, enhances T cell infiltration, disrupting tumor growth in vivo [75]. Furthermore, in Endo180 KO mice, treatment with either anti-CTLA4 or anti-PD-L1 inhibits tumor growth and metastasis and improves survival by increasing the TILs content and infiltration to the tumor [75]. In agreement, melanoma patients with elevated Endo180 expression exhibit poorer responses to anti-PD-1 therapy [75].

A different strategy focused on targeting CAFs was designed by Ford et al. [74]. By inhibiting NOX4, a reactive oxygen species (ROS)-producing enzyme overexpressed in CAFs, authors reduced CAF activity, which resulted in the induction of a quiescent state that promotes T cell infiltration and restores sensitivity to immunotherapy in breast, lung and colorectal mice models [74]. These examples serve as a proof of concept that CAF targeting directly affects immune exclusion, and, thus, it is worth designing additional strategies for such a goal.

As discussed above, stromal cells are an important source of soluble signals that affect both the ECM remodeling and immune cell infiltration. Thus, targeting soluble mediators is also a strategy that has been exploited. For example, FGFR blockade by Erdafitinib suppressed CAF proliferation, migration, and VCAM-1 secretion by downregulating the MAPK/ERK pathway, leading to improved antitumor efficacy of an immune checkpoint therapy [114]. In mouse models of mammary and colorectal carcinoma, coadministration of an anti-TGF-*β* agent along with anti-PD-L1, inhibited TGF-*β* activation in stromal cells and increased T cell infiltration, particularly CD8^+^ T cells, reducing tumor burden [82]. In the breast cancer model, the effect was dependent on CD8^+^ T cells [82]. Similar results were observed by Knudson and colleagues, using a bifunctional anti-PD-L1/TGFβ trap fusion protein (M7824) in mouse breast and colon carcinoma models. This fusion protein is composed of the C-terminus of anti-PD-L1 heavy chain linked to the extracellular domain of TGFβRII. Treatment reduced tumor volume, promoted by an increase in CD8^+^ T and NK cell activation, and improved the overall survival with better efficacy than single therapies targeted to either TGF-*β* or PD-L1 [107]. Moreover, there are ongoing clinical trials for TGF-*β*-targeting therapies. The TGF-*β* 1/3 trap AVID200 is being evaluated in patients with advanced solid tumors (Phase 1) [106]. Bintrafusp alfa is a bifunctional fusion protein directed to TGF-*β* and PD-L1 [107] that has reached Phase 1 trials for advanced solid tumors, showing antitumor activity and safety [115].

To target the signals impairing immune infiltration, Zhao and colleagues enhanced CAR-T cell therapy by developing a nanogel crosslinked with collagenase and a CXCR4 antagonist peptide (DV1). The strategy was tested in murine models of pancreatic cancer. These nanogels are formed when collagenase is oxidized with sodium alginate, which results in a crosslinked collagenase. Then, these nanoplatforms are chemically modified with DV1. DV1 allows the binding to CAR-T cells and, furthermore, impairs the CXCL12/CXCR4 axis. This strategy combines the disruption of ECM barriers with avoidance of stromal retention of T cells by targeting the CXCL12/CXCR4 axis [88].

**Figure 1:**
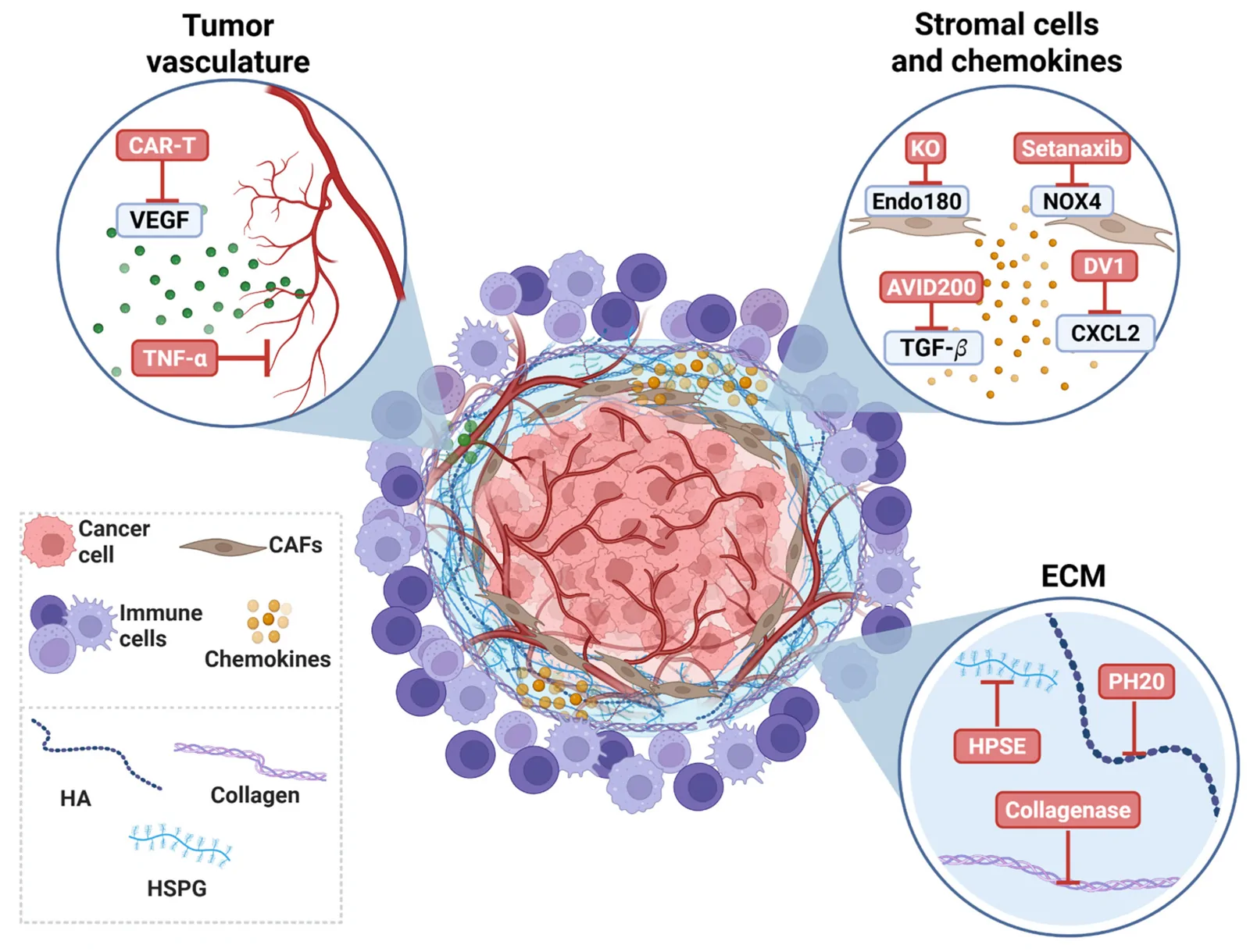
Mechanisms of immune exclusion and emerging therapeutic targets. Schematic illustration of the major components of the TME that drive immune exclusion and limit antitumor immune responses. Immune cells are retained within the peritumoral stroma and fail to efficiently infiltrate the tumor core due to coordinated physical and chemical barriers established by the TME. Three principal mechanisms are highlighted: (i) abnormal tumor vasculature, characterized by VEGF- and TNF-α–regulated pathways that impair leukocyte trafficking and endothelial adhesion; (ii) stromal cells and chemokine secretion, including CAFs, TGF-*β* signaling, CXCL12-mediated immune cell retention, Endo180, and NOX4-dependent stromal activation; and (iii) ECM remodeling, involving collagen deposition, HA, HSPGs, and ECM stiffness, which collectively generate a dense structural barrier that restricts immune cell migration. Representative emerging therapeutic strategies targeting these mechanisms to overcome immune exclusion and enhance antitumor immunity are illustrated, including antiangiogenic approaches, CAR-T-based therapies, TGF-*β* inhibitors, CXCL12/CXCR4 blockade, collagenase, PH20 hyaluronidase, and HPSE-mediated ECM degradation. Created with Biorender Alvarez-Lorenzo, S. (2026). BioRender.com/yhwj7a1 (accessed 11 July 2025).

Altogether, these findings support the concept that stromal remodeling and the neutralization of CAF-mediated suppressive networks are critical steps for enhancing immune cell infiltration and improving the efficacy of cancer immunotherapy. Furthermore, the combination of enzymes degrading ECM and molecules targeting the chemokine-mediated control of immune cell infiltration may have a synergistic antitumor effect (Figure 1).

## 4. Conclusions

Immune exclusion is recognized as a defining hallmark of resistance to antitumor immunity and immunotherapy in solid tumors. Rather than representing a passive absence of immune infiltration, immune exclusion results from an active and coordinated remodeling of the TME involving abnormal vasculature, ECM remodeling, CAF activation, and chemokine dysregulation. Together, these mechanisms generate a highly organized stromal barrier that spatially segregates immune cells from tumor nests and suppresses effective antitumor immunity.

The recent advances reviewed and discussed here demonstrate that immune exclusion can be therapeutically targeted. Targeting vascular anergy, collagen organization, ECM crosslinking, hyaluronan accumulation, CAF activity, and chemokine signaling can partially restore immune cell infiltration and potentiate the efficacy of immune checkpoint blockade and adoptive cellular therapies in preclinical models. To achieve clinical translation, these findings must be supported by additional evidence of their safety, and by the identification of relevant biomarkers that allow patient stratification. Currently, several of these approaches are progressing into clinical evaluation, supporting their translational potential. The emergence of engineered immune cells, multifunctional nanoplatforms, and bacteria-based delivery systems further expands the therapeutic possibilities for stromal remodeling.

The complexity and heterogeneity of the TME remain major obstacles for clinical translation. TME varies considerably among tumor types and even among patients with the same malignancy. Future efforts should focus on identifying robust biomarkers that define immune-excluded phenotypes, determining the optimal timing for stromal-targeting interventions, and understanding the context-dependent roles of stromal components in tumor progression. Emerging technologies such as spatial transcriptomics, multiplex imaging, and single-cell analyses will likely accelerate the characterization of immune-excluded tumors and facilitate precision-based therapeutic interventions.

Another important consideration is that the stromal compartment may also exert tumor-restraining functions under certain contexts. Consequently, excessive stromal depletion or indiscriminate ECM degradation could produce adverse effects, including enhanced tumor dissemination or tissue damage. Future therapeutic strategies should prioritize stromal normalization and immune reprogramming over indiscriminate tissue disruption.

Overall, targeting immune exclusion represents a promising opportunity to convert poorly infiltrated tumors into inflamed and immunologically responsive ones. A deeper understanding of the molecular and biomechanical mechanisms governing immune cell trafficking within tumors will likely enable the development of more effective combinatorial therapies and broaden the clinical benefit of cancer immunotherapy.

## Figures and Tables

**Table 1:** Proportion of immune-excluded tumors across different cancer types *.

Cancer Type	Fraction of Immune-Excluded Tumors
NSCLC	40% [12]
SCLC	64% [12]
Pancreatic cancer	46% [13]
CRC	75% [12]
Ovarian cancer	45% [12]
ccRCC	24% [12]
HCC	19% [14]
Breast cancer	38% [15]
Melanoma	30% [16]

* The reported frequencies come from different studies and may therefore not be directly comparable.

**Table 3:** Therapeutic approaches directed at TME components.

TME Component	Target	Strategy	Phase of Development
Tumor vasculature	VEGF/VEGFR	CAR-T cells targeting VEGF [92]	In vivo
		Anti-VEGF/VEGFR2 agent combined with ex vivo T cells armed with tumor-targeting antibodies [93]	In vivo
	TNF-α	Delivery of low doses of TNF-α and combination with ACT and ICB [29,90,91]	In vivo
ECM	Collagen	Monoclonal antibody against DDR1 [97,98]	Phase 1 trial (NCT05753722)
		LOX inhibition [99]	In vivo
		Probiotic-nanosystem, either with a MMP2-responsive peptide or collagenase [89,104]	In vivo
	HSPG	HPSE [43]	In vivo
		CAR-T cells expressing HPSE [65]	In vivo
		NK cells expressing HPSE [105]	In vivo
	HA	Adenovirus with T cell engager and PH20 [100]	In vivo
		Hyaluronidase	Multiple Phase 1 clinical trials (NCT03656718, NCT02563548, NCT03267940, NCT02346370, NCT01928030, NCT03467867, NCT03481920 NCT00834704, NCT01959139, NCT01839487, NCT05296798, NCT06698042, NCT05722015, NCT02753595, NCT06212752, NCT01170897)
Stromal cells and chemokine secretion	CAFs	Knockout of Endo180 [75]	In vivo
		Targeting NOX4 (Setanaxib) [74]	In vivo
	CXCL12/CXCR4 axis	Nanogel of crosslinked collagenase and DV1 [88]	In vivo
	TGF-*β*	Anti-TGF-*β* plus anti-PDL1 [82]	In vivo
		Anti-PD-L1/TGFβ Trap fusion protein (M7824) [78]	In vivo
		AVID200, a TGFβ 1/3 trap [106]	Phase 1 trial (NCT03834662)
		Bintrafusp Alfa, a bifunctional fusion protein directed to TGF-*β* and PD-L1 [107]	Phase 1 trial (NCT02517398)

## Data Availability

No new data were created or analyzed in this study.

## Abbreviations

The following abbreviations are used in this manuscript:

ACT	Adoptive cell therapy
CAFs	Cancer-associated fibroblasts
CAR	Chimeric antigen receptor
DDR	Discoidin domain-containing receptor
EC-BM	Endothelial basement membrane
ECM	Extracellular matrix
FGFs	Fibroblast growth factors
HPSE	Heparanase
HSPG	Heparan sulfate proteoglycans
ICAM1	Integrin ligands intercellular adhesion molecule 1
ICB	Immune checkpoint blockade
LOX	Lysyl oxidases
MAdCAM-1	Mucosal vascular addressin cell adhesion molecule 1
POSTN	Periostin
PDGF-B	Platelet-derived growth factor-B
TIME	Tumor immune microenvironment
TILs	Tumor-infiltrating lymphocytes
TNBC	Triple-negative breast cancer
TNF-α	Tumor necrosis factor-α
TME	Tumor microenvironment
VEGFs	Vascular endothelial growth factors
VCAM1	Vascular cell adhesion protein 1
